# Identifying tuberculous pleural effusion using artificial intelligence machine learning algorithms

**DOI:** 10.1186/s12931-019-1197-5

**Published:** 2019-10-16

**Authors:** Zenghua Ren, Yudan Hu, Ling Xu

**Affiliations:** Department of Respiratory Medicine, Shanghai Jiao Tong University Affiliated Sixth People’s Hospital, No. 600, YiShan Road, Shanghai, 200233 China

**Keywords:** Tuberculous pleural effusion, Diagnostic model, Artificial intelligence, Machine learning algorithm

## Abstract

**Background:**

The differential diagnosis of tuberculous pleural effusion (TPE) is challenging. In recent years, artificial intelligence (AI) machine learning algorithms have started being used to an increasing extent in disease diagnosis due to the high level of efficiency, objectivity, and accuracy that they offer.

**Methods:**

Data samples on 192 patients with TPE, 54 patients with parapneumonic pleural effusion (PPE), and 197 patients with malignant pleural effusion (MPE) were retrospectively collected. Based on 28 different features obtained via statistical analysis, TPE diagnostic models using four machine learning algorithms (MLAs), namely logistic regression, k-nearest neighbors (KNN), support vector machine (SVM) and random forest (RF) were established and their respective diagnostic performances were calculated. The respective diagnostic performances of each of the four algorithmic models were compared with that of pleural fluid adenosine deaminase (pfADA). Based on 12 features with the most significant impacts on the accuracy of the RF model, a new RF model was designed for clinical application. To demonstrate its external validity, a prospective study was conducted and the diagnostic performance of the RF model was calculated.

**Results:**

The respective sensitivity and specificity of each of the four TPE diagnostic models were as follows: logistic regression – 80.5 and 84.8%; KNN– 78.6 and 86.6%; SVM – 83.2 and 85.9%; and RF – 89.1 and 93.6%. The sensitivity and specificity of pfADA were 85.4 and 84.1%, respectively, at the best cut-off value of 17.5 U/L. RF was the superior method among the four MLAs, and was also superior to pfADA. The newly designed RF model (based on 12 out of 28 features) exhibited an acceptable performance rate for the diagnosis of TPE with a sensitivity and specificity of 90.6 and 92.3%, respectively. In the prospective study, its sensitivity and specificity were 100.0 and 90.0%, respectively.

**Conclusions:**

Establishing a model for the diagnosis of TPE using RF resulted in a more effective, economical, and faster diagnostic method. This method could enable clinicians to diagnose and treat TPE more effectively.

## Background

Tuberculous pleurisy is a common disease that causes pleural effusion. In 2014, approximately 1.5 million tuberculosis patients died worldwide [1]. Accurate diagnosis and timely treatment are vital. The gold standard in the diagnosis of tuberculous pleural effusion (TPE) derives from positive findings in pathogenic and pathological examinations. However, pathogenic diagnosis using smears or cultures of specimens of respiratory tract or pleural fluid exhibits low positivity rates and/or long culturing times [2]. Pathological diagnosis via thoracoscopic pleural biopsy is traumatic, holds the risk of complications, and is associated with high costs and prohibitive technical constraints. Therefore, the diagnosis of TPE remains challenging. In clinical practice, the most widely used diagnostic biomarker for TPE is pleural fluid adenosine deaminase (pfADA). When lymphocyte predominates in exudative pleural fluid with elevated levels of pfADA and no evidence of other diseases, pleural effusion is diagnosed as TPE. However, neutrophils may also predominate during the early stages of TPE [3], and the pfADA cut-off values for the diagnosis of TPE differ across several different studies [4, 5]. Therefore, it is necessary to develop a method for the early diagnosis of TPE which is less invasive and more accurate.

In recent years, research into the use of artificial intelligence (AI) in the field of medicine has increased. Machine learning is a type of AI that allows computers to learn without being explicitly programmed for a given task. Using machine learning algorithms (MLAs) such as support vector machine (SVM), k-nearest neighbor (KNN), and random forest (RF), highly efficient, objective, and accurate disease diagnosis models can be constructed. Based on structural MRI data, Bisenius et al. [6]applied the SVM method for predicting primary progressive aphasia subtypes. Their results showed that the method provided a high degree of accuracy of between 91 and 97%. Forghani et al. [7] used the RF method to design a model for predicting lymph node metastasis of squamous cell carcinoma of the head and neck, achieving a diagnostic accuracy of 88%. Kim et al. used decision tree, RF, KNN and SVM methods to construct several models for the diagnosis of glaucoma [8]. The sensitivity, specificity, and accuracy of these four models reached 95% and higher. The application of MLAs in the diagnosis of TPE is uncommon [9], and comparisons between the diagnostic performances of various algorithmic models have not been drawn. The diagnostic performances of pfADA and MLAs have also not been compared.

In this study, we selected logistic regression, KNN, SVM, and RF to construct TPE diagnostic models. By comparing the respective diagnostic performances of these four models, the most effective model was selected for the differential diagnosis of TPE. We also compared the diagnostic performances of pfADA versus the four MLA methods.

## Methods

### Subjects and study design

Data from patients diagnosed with TPE, parapneumonic pleural effusion (PPE), and malignant pleural effusion (MPE) who had undergone thoracentesis between January 2003 and August 2018 were retrospectively collected.

TPE diagnosis was confirmed when pleural effusion exhibited exudativity and met at least one of the following conditions [10–12]: (1) positive smear for acid-fast bacilli in pleural fluid/sputum/bronchial aspirate/bronchoscopic brushing specimen; (2) positive culture or positive polymerase chain reaction (PCR) for *Mycobacterium tuberculosis* in pleural fluid/sputum/bronchial aspirate; (3) epithelioid caseous granuloma or positive acid-fast staining in pleural or lung tissue; (4) moderately or strongly positive 5 U tuberculin skin test, positive T-cell spot test (T-SPOT), or positive M. tuberculosis antibody test, and a clinical response to anti-tuberculosis treatment; (5) typical symptoms of tuberculosis with no evidence of additional respiratory diseases, and a marked response to anti-tuberculosis treatment. A clinical response to anti-tuberculosis treatment refers to symptomatic relief, remission or elimination of pleural effusion in patients who have been followed up for at least 12 months after receiving anti-tuberculosis treatment.

MPE was diagnosed if pleural effusion was exudative and met one of the following criteria [11]:(1) malignant cells were found in lung tissue; (2) malignant cells were found in pleural fluid or pleural tissues.

PPE was diagnosed if patients met all the following criteria [11]: (1) exudative effusion associated with pneumonia; (2) absence of other causes of pleural effusion; (3) the patient’s symptoms disappeared, lung shadows and pleural effusion were absorbed after a two-month follow-up after antibiotic treatment.

The exclusion criteria were as follows: (1) patients with transudative pleural effusion; (2) patients without pfADA results; (3) patients in the TPE and PPE groups who were unable to provide information during follow-up visits.

The following features were evaluated: patients’ gender and age, symptoms (fever, cough, sputum, bloody sputum, chest tightness, chest pain, anorexia, fatigue, night sweats, weight loss), history of smoking, hematologic parameters (total and differential cell count, erythrocyte sedimentation rate (ESR), C-reactive protein (CRP), ADA, lactate dehydrogenase (LDH), carcinoembryonic antigen (CEA)), pleural fluid parameters (bloody effusion, Rivalta test, total and differential cell count, total protein, glucose, chloride, ADA, LDH, and CEA concentrations). In cases where more than one thoracocentesis had been performed, the statistical analysis was performed using only the data from the first pleural fluid sample prior to commencing treatment. Hematological data were obtained from the blood samples taken nearest to the first thoracentesis.

ADA activity was measured using enzymatic colorimetry (ADA kit, Junshi Biotechnology Co., Ltd., Shanghai, China). LDH levels were measured using the lactic acid substrate method (LDH assay kit, DiaSys Diagnostic Systems Shanghai Co., Ltd., Germany). CRP levels were measured via scattering immunoturbidimetry (Lifotronic PA-900 specific protein analyzer and original reagent, Shenzhen, China). CEA levels were measured using chemiluminescent immunoassay kits (Roche, Mannheim, Germany). ESR was determined using an Italian TEST1TH Automatic Blood Sedimentation Instrument. Specific gravity was measured via dry chemical analysis (American iChem VELOCITY Automatic Analyzer). Total and differential cell counts in blood and total red/white blood cells in pleural fluid were measured using a Japanese Sysmex XN-3000 Automatic Blood Analyzer. Differential cell counts in pleural fluid were counted manually. Total protein, glucose, and chloride concentrations were measured using an Hitachi 7600 Automatic Biochemical Analyzer and original reagents.

### Statistical analysis and the design and evaluation of algorithmic models

Statistical analysis was performed using the SPSS version 20.0 software (SPSS Inc., Chicago, IL, USA). The qualitative variables were presented as numbers and percentages, and the continuous variables were presented as medians and ranges. The differences in qualitative variables between patients with and without TPE were assessed using the Chi-square test. The differences between the continuous variables were analyzed using the Mann-Whitney U Test. Statistical significance was set at *P* < 0.05, and statistically significant variables were introduced into the diagnostic models.

**Fig. 1:**
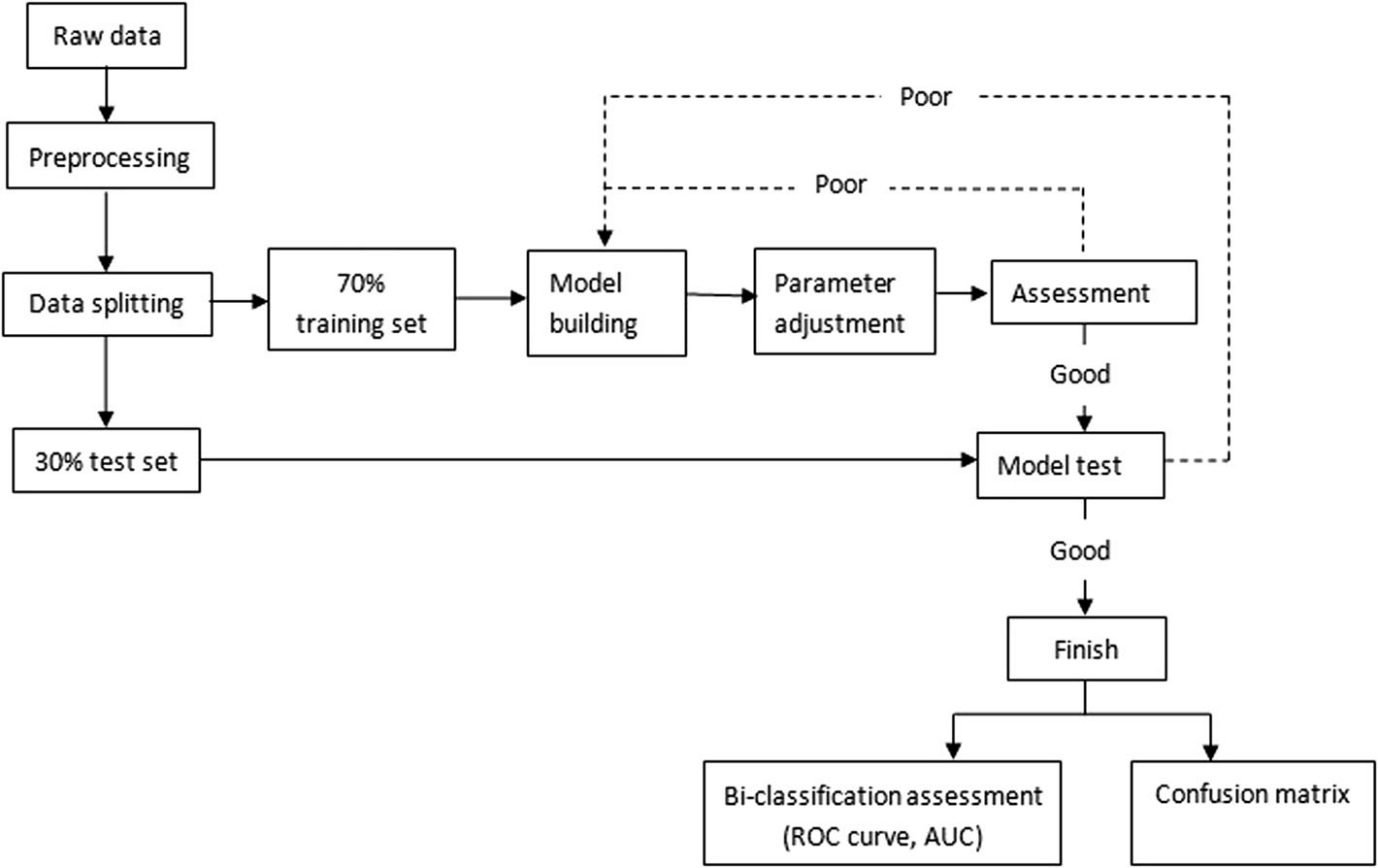
Workflow for constructing diagnostic models using MLAs

For this study, we selected four MLAs to establish models for the diagnosis of TPE, namely logistic regression, KNN, SVM, and RF. The workflow for constructing the models was as follows (Fig. 1):

In the process of establishing the models, logistic regression, KNN, and SVM required data preprocessing. The data were scaled proportionally so that the unit restrictions could be removed without changing the original data distribution, and the missing values were set to the normalized average values. The RF model did not require data preprocessing, and the original data were input directly for splitting.

The four methods’ parameters were set as follows: (1) for logistic regression, the maximum number of iterations was 100, the regularization coefficient was 1, and the minimum convergence error was 0.000001; (2) for KNN, the nearest neighbor number was 5; (3) for SVM, the positive penalty factor was 1.0, the negative penalty factor was 1.0, and the convergence coefficient was 0.001; (4) for RF, the number of trees in the forest was 100, the minimum amount of leaf node data was 2, the minimum proportion of leaf node data to parent node data was 0, the maximum depth of a single tree was infinite, and the amount of random data input by a single tree was 100,000.

The diagnostic performances of the four algorithmic models (i.e., sensitivity, specificity, and accuracy) were calculated based on the confusion matrix which included the predicted and actual classification data. Since the data were randomly allocated to a 70% training set and a 30% test set, we conducted 20 model-building tests on each algorithm to determine the average sensitivity, specificity, positive predictive value (PPV), negative predictive value (NPV), positive likelihood ratio (PLR), negative likelihood ratio (NLR), and accuracy of each of the four algorithmic models as the final results. The best cut-off value and diagnostic performance of pfADA were assessed using receiver operating characteristic (ROC) curve analysis.

Among the four algorithmic models, we found that RF offered the best diagnostic performance. The respective impacts of each feature on the accuracy of the RF model were rank ordered. In the RF model, larger Gini index average reduction values indicated a greater effect of a particular feature on the accuracy of the classification model.

## Results

### Patient characteristics

During the study, 1262 patients with pleural effusion were reviewed, of which 819 were excluded from the analysis for the following reasons: (1) 188 patients exhibited transudative pleural effusion; (2) 213 patient had not undergone thoracentesis; (3) 10 patients lacked pfADA results; (4) 179 patients in the TPE and PPE group were unable to provide information during follow-up visits; (5) 229 patients exhibited pleural effusion with unclear causes. Finally, 443 patients met the analysis criteria, namely 192 with TPE, 54 with PPE, and 197 with MPE. The patients’ malignancies associated with pleural effusion were as follows: lung cancer (172 patients); pleural mesothelioma (3 patients); breast cancer (5 patients); lymphoma (2 patients); pancreatic cancer (1 patient); gastric cancer (3 patients); duodenal ampullary carcinoma (1 patient); thyroid cancer (5 patients); pleural bidirectional differentiation malignant tumor (epithelioid hemangioendothelioma) (1 patient); laryngeal carcinoma (1 patient); papillary carcinoma of the nasal cavity (1 patient); adenocarcinoma with an unknown primary site (2 patients).

### Disease characteristics model criteria

Among the 36 features, 28 differed significantly between TPE and non-TPE patients (Table 1). The following 28 features were introduced into the model: age, fever, cough, chest pain, anorexia, fatigue, night sweats, history of smoking, total blood WBC, neutrophil percentage (N%) in blood, lymphocyte percentage (L%) in blood, monocyte percentage (M%) in blood, platelet count (PLT), ESR, CRP, serum LDH, serum ADA, serum CEA, bloody effusion, Rivalta test results, total WBC in pleural fluid, N% in pleural fluid, L% in pleural fluid, pleural fluid total protein, pleural fluid glucose concentration, pleural fluid LDH (pfLDH), pfADA, pleural fluid CEA (pfCEA).

**Table 1:** Comparison of clinical and laboratory findings between TPE and non-TPE patients

	TPE(*n* = 192)	non-TPE(*n* = 251)	*P* value
Gender			
Male	131(68.2)	157(62.5)	0.214
Female	61(31.8)	94(37.5)	
Age(years)	36.5(24.3, 59.0)	67.0(56.0, 77.0)	0.000
Has a history of smoking	53(27.6)	96(38.2)	0.019
Fever> 37.5 °C	127(66.1)	57(22.7)	0.000
Cough	151(78.6)	205(81.7)	0.011
Sputum	86(44.8)	143(57.0)	0.427
Bloody sputum	2(1.0)	12(4.8)	0.526
Chest tightness	73(38.0)	117(46.6)	0.070
Chest pain	106(55.2)	103(41.0)	0.003
Anorexia	68(35.4)	98(39.0)	0.000
Fatigue	57(29.7)	24(9.6)	0.000
Night sweats	60(31.3)	12(4.8)	0.000
Weight loss	32(16.7)	40(15.9)	0.700
In blood:			
WBC(× 10^9^/L)	5.8(4.9, 7.3)	7.6(5.8, 9.9)	0.000
N%	66.4(60.0, 72.4)	71.3(65.8, 77.3)	0.000
L%	19.8(15.4, 24.2)	18.0(12.4, 23.4)	0.009
M%	10.5(8.5, 13.1)	7.7(6.0, 9.2)	0.000
HB(g/L)	129.0(119.0, 139.0)	130.0(116.5, 140.5)	0.564
PLT(×10^9^/L)	281.0(227.3, 342.5)	250.0(210.0, 301.8)	0.000
ESR(mm/h)	59.0(43.0, 85.0)	36.0(19.0, 69.0)	0.000
CRP(mg/L)	58.8(29.8, 101.0)	26.6(6.7, 76.2)	0.000
LDH(U/L)	191.5(158.3, 225.8)	208.0(171.0, 280.0)	0.044
ADA(U/L)	9.0(7.0, 12.0)	7.0(5.0, 11.0)	0.022
CEA(ng/mL)	1.4(0.9, 2.2)	5.9(2.1, 29.1)	0.000
In pleural fluid:			
Bloody effusion	2(1.0)	40(15.9)	0.000
Positive Rivalta test	189(98.4)	212(84.5)	0.000
WBC(× 10^6^/L)	1200.0(427.3, 2560.0)	432.5(135.0, 1200.0)	0.000
RBC(× 10^6^/L)	1600.0(800.0, 3160.0)	1600.0(720.0, 4420.0)	0.218
N%	10.0(4.0, 21.0)	19.0(6.0, 50.0)	0.000
L%	86.5(70.0, 92.0)	70.0(41.0, 86.0)	0.000
Total protein(g/L)	52.0(49.0, 55.0)	47.0(42.0, 52.0)	0.000
Glucose(mmol/L)	5.2(4.4, 6.5)	6.1(4.4, 7.2)	0.004
Chloride(mmol/L)	104.0(100.3, 106.0)	104.0(101.0, 107.0)	0.089
LDH(U/L)	415.0(265.0, 609.0)	264.0(167.0, 460.0)	0.000
ADA(U/L)	26.0(21.0, 40.0)	7.0(5.0, 13.0)	0.000
CEA(ng/mL)	1.0(0.6, 1.6)	30.0(1.9, 170.0)	0.000

Data in the table are expressed either as a frequency (percentage) or a median (interquartile range)

*TPE* tuberculous pleural effusion, *non-TPE* non-tuberculous pleural effusion (including parapneumonic pleural effusion and malignant pleural effusion), *WBC* white blood cells, *RBC* red blood cells, *N* neutrophils, *L* lymphocytes, *M* monocytes, *HB* hemoglobin, *PLT* platelets, *ESR* erythrocyte sedimentation rate, *CRP* C-reactive protein, *LDH* lactate dehydrogenase, *ADA* adenosine deaminase, *CEA* carcinoembryonic antigen

### Diagnostic performances of pfADA and the four algorithmic models

**Fig. 2:**
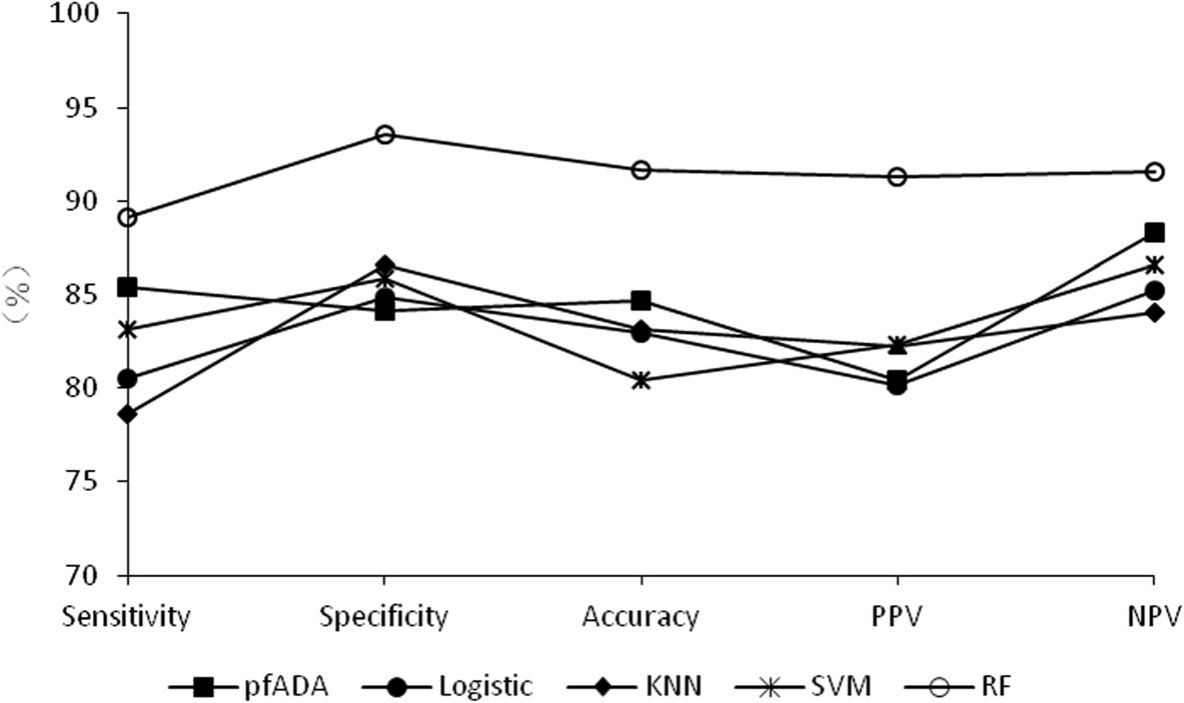
Sensitivity, specificity, accuracy, PPV, NPV curves for pfADA and the four algorithmic models. PPV: positive predictive value; NPV: negative predictive value; pfADA: pleural fluid adenosine deaminase; Logistic: logistic regression; KNN: k-nearest neighbor; SVM: support vector machine; RF: random forest

**Fig. 3:**
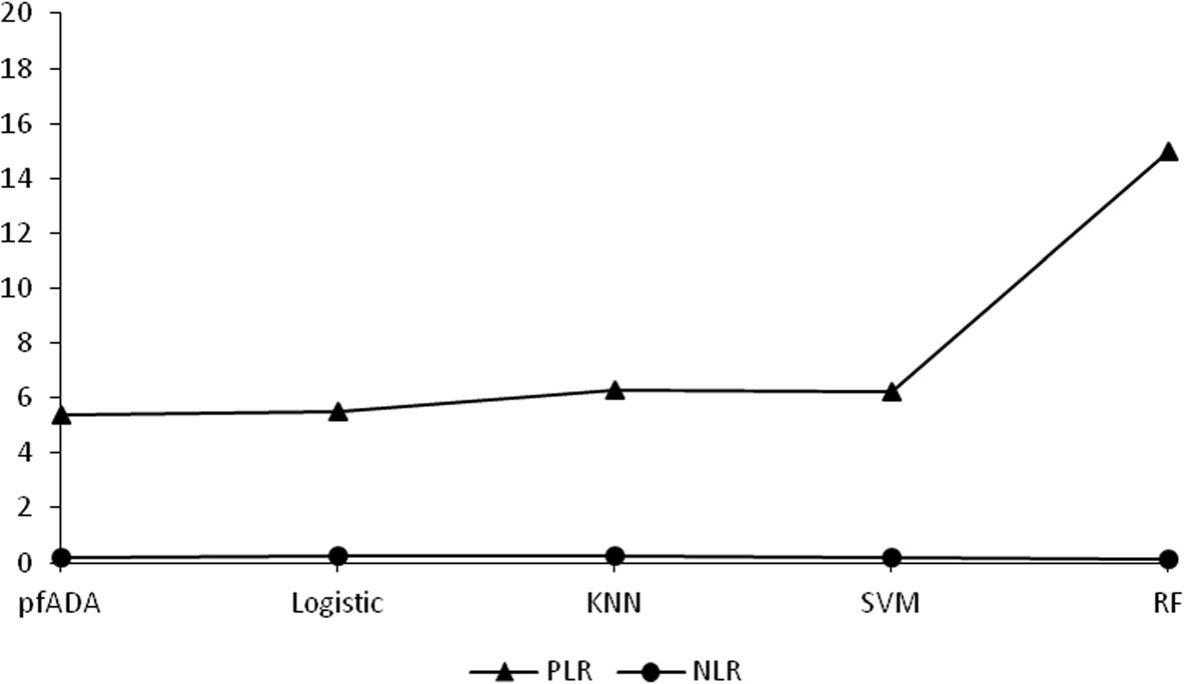
The PLR and NLR curves for pfADA and the four algorithmic models. PLR: positive likelihood ratio; NLR: negative likelihood ratio; pfADA: pleural fluid adenosine deaminase, Logistic: logistic regression; KNN: k-nearest neighbor; SVM: support vector machine; RF: random forest

The best cut-off value for pfADA in the diagnosis of TPE was 17.5 U/L with a sensitivity of 85.4%, a specificity of 84.1% and an accuracy of 84.7%. The TPE diagnostic performances of the four algorithmic models and pfADA are presented in Table 2, Fig. 2 and 3. Among the four algorithmic models, RF is the superior method for diagnosing TPE, with a sensitivity, specificity, PPV, NPV, PLR, and accuracy higher than those of logistic regression, KNN, SVM, and pfADA. NLR was lower than pfADA and the other three algorithmic models.

**Table 2:** Performances of the four algorithmic models and pfADA for diagnosing TPE

	AUC	Sensitivity	Specificity	PPV	NPV	PLR	NLR	Accuracy
pfADA	0.890	85.4%	84.1%	80.4%	88.3%	5.37	0.17	84.7%
Logistic regression	0.876	80.5%	84.8%	80.2%	85.2%	5.47	0.23	82.9%
KNN	0.895	78.6%	86.6%	82.3%	84.0%	6.28	0.24	83.2%
SVM	0.918	83.2%	85.9%	82.3%	86.6%	6.23	0.20	80.4%
RF	0.971	89.1%	93.6%	91.3%	91.5%	14.97	0.12	91.6%

*TPE* tuberculous pleural effusion, *pfADA* pleural fluid adenosine deaminase, *KNN* k-nearest neighbor, *SVM* support vector machine, *RF* random forest, *AUC* area under the curve, *PPV* positive predictive value, *NPV* negative predictive value, *PLR* positive likelihood ratio, *NLR* negative likelihood ratio

### The impact of each feature on the accuracy of the RF model

According to the Gini index average reduction values, the respective impact of each feature on the accuracy of the RF model (from high to low) were as follows: pfADA, pfCEA, age, total blood WBC, M% in blood, L% in pleural fluid, N% in pleural fluid, fever, night sweats, pleural fluid total protein, pfLDH, serum CEA, total WBC in pleural fluid, ESR, glucose concentrations in pleural fluid, platelet count, CRP, N% in blood, fatigue, bloody pleural fluid, L% in blood, serum LDH, Rivalta test results, serum ADA, chest pain, history of smoking, anorexia, and cough.

**Fig. 4:**
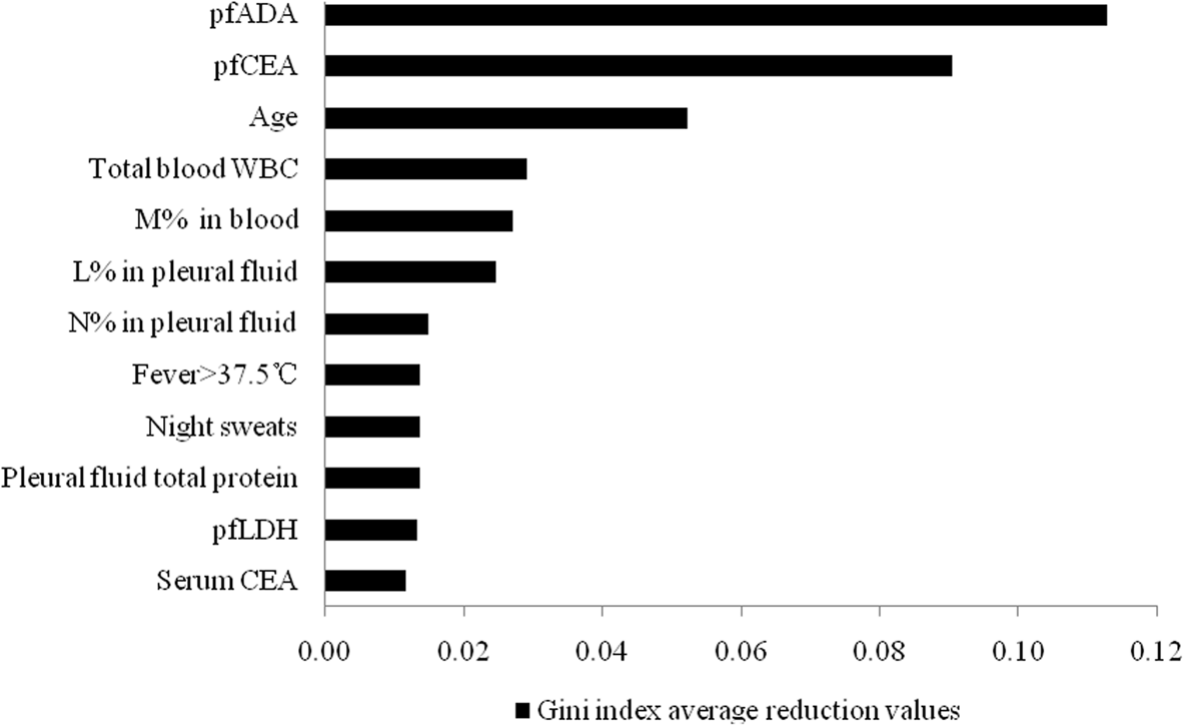
The impacts of the first 12 features on the accuracy of the RF model. pfADA: pleural fluid adenosine deaminase; pfCEA: pleural fluid carcinoembryonic antigen; WBC: white blood cells; M: monocyte; L: lymphocyte; N: neutrophil; pfLDH: pleural fluid lactate dehydrogenase

Since the lower-ranking features had negligible impacts on the accuracy of the classification model, the Gini index average reduction values are only given for the first 12 features (Fig. 4).

### Diagnostic performance of the new RF model using the first 12 features

To reduce the number of features used in the RF model for the sake of clinical application, we selected 12 features to establish the new RF model. The process was the same as in Fig. 1. The diagnostic performance of the new RF model for the diagnosis of TPE is shown in Table 3.

**Table 3:** Performance of the new RF model for diagnosing TPE

	AUC	Sensitivity	Specificity	PPV	NPV	PLR	NLR	Accuracy
RF	0.965	90.6%	92.3%	80.9%	93.0%	13.1	0.1	91.5%

*RF* random forest, *TPE* tuberculous pleural effusion, *AUC* area under the curve, *PPV* positive predictive value, *NPV* negative predictive value, *PLR* positive likelihood ratio, *NLR* negative likelihood ratio

### Diagnostic performance of the RF model for TPE in prospective study

To demonstrate external validity of our study, we prospectively collected data from 27 patients with pleural effusion from October 2018 to August 2019. The patients were 18 to 86 years old and the median age was 66 years old. 15 patients (55.6%) were female. Using our RF model, 9 patients were diagnosed as TPE and 18 patients were diagnosed as non-TPE. After a series of examinations, the final confirmed diagnosis was as follows: 7 cases of TPE (5 cases with epithelioid caseous granuloma in pleural tissue, 1 case with epithelioid caseous granuloma in lung tissue, 1 case with positive PCR for *Mycobacterium tuberculosis* in bronchial aspirate), 20 cases of non-TPE (3 cases of PPE, 17 cases of MPE). Compared with the final diagnosis, only 2 cases were misdiagnosed as TPE (1 case of PPE and 1 case of MPE). The sensitivity, specificity and accuracy for diagnosing TPE were 100.0, 90.0, and 92.6%, respectively.

## Discussion

AI has heralded changes in all aspects of society in recent years, and research into its potential uses in the medical field is also expanding. In disease diagnosis, AI machine learning algorithms can process vast amounts of data while making the best use of preexisting information to develop highly predictive disease diagnostic models. Presently, MLAs used in medical diagnostics include logistic regression, KNN, SVM, RF and others. Hwang et al. developed a deep-learning-based automatic detection (DLAD) algorithm for detecting active pulmonary tuberculosis on chest radiographs. The study results showed that DLAD is superior to thoracic radiologists in terms of image classification and lesion localization [13].

Logistic regression is a classical statistical method. The output result of the logistic regression model is a probabilistic value of 0 to 1, with a value of 0.5 indicating a double classification. Logistic regression is widely used in medical diagnostics. For example, based on pfADA, Interferon-γ (IFN-γ), decoy receptor (DcR) 3 and soluble tumour necrosis factor receptor 1 (TNF-sR1) measurements, Shu et al. [14] constructed a logistic regression model for the diagnosis of TPE with a sensitivity of 82.9% and a specificity of 86.7%. Gonzalez et al. [15] also used logistic regression to construct a model to differentiate TPE from MPE with a diagnostic sensitivity of 93.5% and a specificity of 78%. However, DcR3, TNF-sR1, and IFN-γ are not routinely measured clinically, meaning that the model designed by Shu et al. is not broadly applicable. In the study of shu, the logistic regression model offers a higher degree of sensitivity compared to that of pfADA, but with a lower degree of specificity (98.3% versus 86.7%). The study conducted by Gonzalez et al. included only 47 TPE patients and 25 MPE patients. As a result of this small number of case subjects and the exclusion of pfADA, CEA, hematological parameters, and patients’ symptoms in the analytical model, it offered a low degree of specificity. Therefore, both models mentioned above have marked shortcomings.

The decision process involved in the KNN method consists of a majority vote. When a test sample is input, the voting occurs based on the categories included in the k-nearest training samples, and the test sample is categorized according to the category with the largest number of votes. Chen et al. [16] applied the KNN method to distinguish normal respiratory sounds from abnormal respiratory sounds. In an ideal sonic environment with no human interference, the method achieved a 100% discrimination rate. Although the study is yet to be duplicated in a realistic sonic environment, it showed that the KNN method holds great potential.

SVM is another MLA that can be used for classification. SVM principally works by constructing a hyperplane to maximize the distance between two types of samples and the hyperplane. Levman et al. [17] used the SVM method to identify malignant and benign breast lesions based on vascular heterogeneity data, achieving an average AUC of 0.79. Kanesaka et al. [18] used the SVM method to diagnose early-stage gastrointestinal cancer in magnified narrow-band images with an accuracy of 96.3%.

RF is another classifying method that comprises multiple decision trees that integrate input information via an If-Then rule to construct a tree classifier. According to the constructed model, new input information is assigned to the leaf nodes via the root node, and the final level result represents the final classification result. However, when the decision tree is excessively deep, over-fitting may occur, potentially resulting in inaccurate results. RF adopts the concept of integrated learning to synthesize the classification results of each decision tree to prevent over-fitting, thus yielding more accurate and stable results. Xiao et al. [19] used the RF method to construct a diagnostic model for prostate cancer with an accuracy of 83.1%, a sensitivity of 65.6%, and a specificity of 93.8%. Casanova et al. [20] compared the diagnostic performances of logistic regression and RF for the diagnosis of diabetic retinopathy. The study’s results demonstrated that RF offers a higher degree of classification accuracy.

At present, few studies have been conducted on the use of AI machine learning alogrithms in the diagnosis of TPE, and no comparisons have been drawn between the diagnostic performance of MLAs and that of pfADA. In this study, we constructed diagnostic models for TPE using four MLAs (logistic regression, KNN, SVM, and RF) and compared the respective diagnostic performances of these four models to select the superior one for the differential diagnosis of TPE. We also compared the diagnostic performance of MLAs versus that of pfADA. 28 features with statistical differences between the TPE group and the non-TPE group were introduced into the model, including age, symptoms, haematological parameters, and pleural fluid measurements. All of these measures are routinely used in clinical practice and were, therefore, quickly and easily obtainable without the need for specialized equipment. Therefore, the models presented in this paper are broadly applicable. The results show that RF exhibits the best diagnostic performance among the four algorithms with a sensitivity, specificity, accuracy and AUC of 89.1, 93.6, 91.6%, and 0.971, respectively. SVM, KNN, and logistic regression exhibited similar diagnostic performances. Previous studies have demonstrated that RF exhibits superior performance for the classification of various diseases. Chen et al. [21] employed four MLAs (SVM, naive Bayes, KNN and RF) to construct decision-support systems in the diagnosis of liver fibrosis. The results indicated that RF provided the highest degree of accuracy among the four MLAs. Chicco et al. [22] compared the performance of probabilistic neural networks, perceptron-based neural networks, RF, One Rule (OneR), and decision tree classifiers in the predictive diagnosis of pleural mesothelioma. Their results showed that RF outperformed all the other MLA models. Therefore, RF is evidently advantageous for the application of disease diagnosis. In this study, pfADA, SVM, KNN, and logistic regression exhibited similar performances in the diagnosis of TPE, while RF stands as the superior method.

To facilitate clinical application, we selected the 12 features with the most significant impacts on the accuracy of the RF model to construct a new RF model. The results show that the diagnostic performance of the new model is similar to that of the RF model constructed with 28 features. Reducing the number of features in the model is highly significant because it may reduce medical expenses and is more convenient in clinical application.

Finally, we conducted a preliminary prospective study to demonstrate external validity of our research. So far, only 27 patients have been enrolled. While small, the current result from our prospective study confirms the validity of our original study. That is to say, RF has high sensitivity, specificity, and accuracy in diagnosing TPE.

The limitations of this study are that the data was sourced from a single center population, and the number of subjects was small. In the future, a multi-center prospective study which includes a large sample size should be conducted to establish a more accurate TPE diagnostic model.

## Conclusions

Using AI machine learning algorithms to establish a model for the diagnosis of TPE may improve diagnostic performance. In this regard, RF is superior to logistic regression, KNN, SVM, and pfADA. Establishing a model for the diagnosis of TPE using RF may provide a more effective, economical, and faster diagnostic method based on routine clinical data to assist clinicians in making better diagnoses and treatment decisions.

## Data Availability

The datasets used or analyzed during the current study are available per reasonable request from the corresponding author.

## Abbreviations

AI: Artificial intelligence
CEA: Carcinoembryonic antigen
CRP: C-reactive protein
DcR: Decoy receptor
DLAD: Deep-learning-based automatic detection
ESR: Erythrocyte sedimentation rate
IFN-γ: Interferon-γ
KNN: K-nearest neighbours
L%: Lymphocyte percentage
LDH: Lactate dehydrogenase
M%: Monocyte percentage
MLAs: Machine learning algorithms
MPE: Malignant pleural effusion
N%: Neutrophil percentage
NLR: Negative likelihood ratio
NPV: Negative predictive value
PCR: Polymerase chain reaction
pfADA: pleural fluid adenosine deaminase
pfCEA: Pleural fluid CEA
pfLDH: pleural fluid LDH
PLR: Positive likelihood ratio
PLT: Platelet count
PPE: Parapneumonic pleural effusion
PPV: Positive predictive value
RF: Random forest
ROC: Receiver operating characteristic
SVM: Support vector machine
TNF-sR1: Soluble tumour necrosis factor receptor 1
TPE: Tuberculous pleural effusion;
T-SPOT: T-cell spot test
